# The Health Effects Of Expanding The Earned Income Tax Credit: Results From New York City

**DOI:** 10.1377/hlthaff.2019.01556

**Published:** 2020-07

**Authors:** Emilie Courtin, Kali Aloisi, Cynthia Miller, Heidi L. Allen, Lawrence F. Katz, Peter Muennig

**Affiliations:** Department of Public Health, Environments, and Society in the Faculty of Public Health and Policy at the London School of Hygiene and Tropical Medicine, in London, United Kingdom.; Department of Statistics, University of Michigan, in Ann Arbor.; Low-Wage Workers and Communities Policy Area, MDRC, in New York City.; School of Social Work, Columbia University, in New York City.; Department of Economics, Harvard University, in Cambridge, Massachusetts.; Mailman School of Public Health, Columbia University.

## Abstract

Antipoverty policies may hold promise as tools to improve health and reduce mortality rates among low-income Americans. We examined the health effects of the New York City Paycheck Plus randomized controlled trial. Paycheck Plus tests the impact of a potential fourfold increase in the Earned Income Tax Credit for low-income Americans without dependent children. Starting in 2015, Paycheck Plus offered 5,968 study participants a credit of up to $2,000 at tax time (treatment) or the standard credit of about $500 (control). Health-related quality of life and other outcomes for a representative subset of these participants (*n* = 3,289) were compared to those of a control group thirty-two months after randomization. The intervention had a modest positive effect on employment and earnings, particularly among women. It had no effect on health-related quality of life for the overall sample, but women realized significant improvements.

In the United States, poverty is associated with a greater burden of disease than smoking and obesity combined.^1^ While there is evidence that socioeconomic factors causally produce poor health,^2,3^ poor health can also lead to unemployment,^4^ bankruptcy,^5^ and impoverishment.^6^ Poor health in early life is also associated with lower educational attainment^7^ and negative outcomes in the labor market,^8^ both of which contribute to the reproduction of income and wealth gaps across generations. Poverty begets poor health and thus more poverty.

The health-poverty trap might be broken with effective antipoverty programs.^9^ The Earned Income Tax Credit (EITC)—a refundable tax credit for low-income workers—has emerged as one such potential policy lever. The policy aims to both reduce poverty and encourage work by providing a refundable credit at tax time to eligible low-income families. By doing so, the EITC could be an effective strategy for improving health.^10^

A characteristic of the EITC and the US welfare system is that Americans without dependent children receive less from antipoverty programs than those with children.^11^ Yet Americans without dependent children have experienced declines in wages^12^ and suffered from widening health disparities over time, compared to adults with incomes above the poverty level.^13^ For people who do have children, the EITC is widely viewed as a successful antipoverty program. It is credited with reducing the number of people in poverty by over 15 percent since its inception, and it has reduced child poverty by over 25 percent.^11^ In addition, the EITC has been shown to encourage work, which has helped it receive bipartisan support.^14^

Despite its popularity, the EITC has not been formally evaluated using randomized controlled trials. Instead, quasi-experimental studies have sought to document its impact on both socioeconomic well-being and health. These studies suggest that the EITC produces positive effects on earnings and income and mixed effects on health. The tax credit and its federal expansions have been associated with better health among mothers and children,^10^ including birth outcomes^15–17^ and physical health and mental health.^18–22^ However, it is also associated with increased obesity and worsened metabolic markers in some studies,^23,24^ but not others.^18^ State supplemental programs have been shown to produce net positive effects on health-related quality of life and survival.^25^

While it appears that children of EITC recipients more clearly benefit than their parents,^22^ little is known about recipients who do not have the additional stressors associated with child rearing. In theory, adults without children could benefit from a generous antipoverty policy such as the EITC because they can work without the expense of child care. However, under the program’s current structure, adults without dependent children are eligible for a maximum credit of just over $500 (about 15 percent of the maximum for one-child families), and it phases out at very low income levels (about $15,000).^11^

The Paycheck Plus demonstration was conceived to address this gap in the US welfare system for adults without dependent children by expanding the credit while also affording an opportunity to evaluate the program using a gold-standard randomized controlled trial. In this article we describe the experiment and its impact on health-related quality of life during its first thirty-two months in New York City.

## The Paycheck Plus Demonstration

Paycheck Plus has been evaluated in New York City by MDRC, a nonprofit social policy evaluation organization. The demonstration is still under way in Atlanta, Georgia. At the New York City site, MDRC partnered with the Mayor’s Office for Economic Opportunity to design and test Paycheck Plus. The two organizations partnered with the New York City Human Resources Administration and the Food Bank for New York City for the program’s implementation.

The 5,968 people who were recruited at baseline were randomly assigned to the treatment group eligible for Paycheck Plus or to a control group whose members were not eligible but could still receive existing tax credits and benefits. The project team conducted substantial out-reach in the months leading up to each tax season to remind participants about their eligibility and the structure of the program (including the maximum bonus they might receive).

In the treatment group, noncustodial parents and workers without qualifying children receive a maximum of about $500 for the federal EITC and lose eligibility once their earnings reach about $15,000. The program tests the effects of a generous expansion of the EITC for that group (see below).

While the Paycheck Plus demonstration operated outside the tax system, it was designed to mirror the process of applying for and receiving the federal EITC. Participants were required to have earned income in the eligible range and to file federal income taxes (for an overview of the recruitment criteria and process, see online appendix exhibit 1).^26^ An important difference was that participants had to apply each year by identifying themselves as Paycheck Plus participants with one of the Food Bank for New York City’s Volunteer Income Tax Assistance workers or by bringing copies of their tax returns to a Volunteer Income Tax Assistance site. Once eligibility for credits was determined, MDRC worked with the Food Bank for New York City to request, issue, and monitor the deposit of each credit to a bank account or debit card. The Paycheck Plus program (the treatment group of the randomized controlled trial) was available for three years, with credits payable at tax time in 2015, 2016, and 2017 based on earnings from the previous year. Paycheck Plus received approval from the MDRC Institutional Review Board.

Participants were recruited between September 2013 and February 2014 (for an an overview of the recruitment criteria and process, see appendix exhibit 1).^26^ Eligibility was based on a combination of family status (single and not planning to claim a dependent child on their tax form), age (ages 21–64; the federal EITC age range for eligibility is 25–64), income (earning less than $30,000 in the prior year), and benefit receipt (not receiving or applying for Supplemental Security Income or Social Security Disability Insurance). Single people who married during the program remained eligible to receive the credit, but those who became parents did not. (The earned income tax credit for workers with dependent children is more generous than Paycheck Plus’s bonus.)

## Study Data And Methods

### DESIGN

Our analysis drew on two rounds of survey data that captured baseline characteristics and health outcomes thirty-two months into the program. Survey data were first collected for all 5,968 participants at study entry, including demographic and socioeconomic characteristics, previous involvement with the criminal justice system, and whether participants had filed income tax returns and received the EITC in the previous fiscal year. For budgetary reasons, a subset of the sample (4,749 people, or 80 percent) was randomly selected and interviewed over the phone approximately thirty-two months after random assignment to one of the study groups, just after the second bonus payment. The survey collected information on employment, earnings, income, housing status, family structure, and health. About 2 percent of the selected subsample were found to be ineligible because of death, incarceration, or lack of fluency in English or Spanish. An additional seventeen participants were not included because of missing consent forms at baseline. The overall response rate was 69 percent (*n* = 3,289), with 72 percent (*n* = 1,701) of the treatment group and 67 percent (*n* = 1,588) of the control group responding (for an overview of the sample selection, see appendix exhibit 2).^26^ Analyses that compared survey respondents and nonrespondents indicated some small significant differences in baseline characteristics, with women and people with higher earnings being more likely to respond to the survey.^27^ However, the survey sample was representative of the full sample. Systematic differences in response rates or missing data were minor and unlikely to bias our assessment of the effect of Paycheck Plus on health.^27^

### HEALTH-RELATED QUALITY OF LIFE

Our outcome of interest was the EQ5D-5L, the most commonly used measure of patient-reported outcomes.^28^ The EQ5D-5L measures five domains of quality of life (mobility, self-care, usual activities, pain/discomfort, and anxiety/depression) on a five-level scale (no problems, slight problems, moderate problems, severe problems, or debilitating problems). The EQ5D-5L has been shown to compare well with similar measures across participants of varying ages and health states.^28^ In addition to serving as a standardized measure of morbidity and disease severity, it can also be used to equate morbidity and mortality. This is accomplished by translating the five-point scale into a scale ranging from 0 to 1 (0 being equal to death and 1 to a state of perfect health). Each domain undergoes testing, using preference weights derived from a large sample of volunteers in the US.^29^ We estimated the effects of the intervention on the overall EQ5D-5L and by domains of the scale.

### APPROACH

Our analysis relied on the experiment’s random assignment to generate unbiased estimates of the effect of expanding the EITC on health-related quality of life.

We first conducted an intent-to-treat analysis to assess the impact of Paycheck Plus on health. This approach examines outcomes for participants in the treatment group relative to those for the control group, irrespective of whether participants actually received the intervention. We used a generalized linear model with a Poisson distribution and log link to improve precision and eliminate any group imbalances. This model adjusts for skewness and heteroscedasticity and approximates the distribution of the outcome data.^30^ We also report in appendix exhibit 3 results from ordinary least squares models.^26^ The results are in the same direction and qualitatively similar to those of our preferred Poisson models. Ordinary least squares models do not account for the skewed distribution of the EQ5D-5L score in our sample (for the distribution of the EQ5D-5L in our sample, see appendix exhibit 4),^26^ and Poisson regressions were consequently preferred.

All models controlled for age, sex, education level, race/ethnicity, earnings in the year before enrollment in Paycheck Plus, history of incarceration, and timing of data collection.

Second, we explored heterogeneous effects of the program on physical health by sex. Results on the effects of Paycheck Plus in New York on socioeconomic outcomes indicated larger positive effects on women’s employment and earnings, compared to men’s.^27,31^ We tested whether the impact of the program on health-related quality of life was also stronger among women by interacting the treatment assignment with sex.

### LIMITATIONS

This study had several limitations. First, only one-third to one-half of the participants received Paycheck Plus in any given year.

Second, although our findings had strong internal validity, the part of the trial reported in this article took place in New York City, and our results are not necessarily representative of those in other cities or states.

Third, the thirty-two-month survey was of a randomly selected subsample (80 percent), which reduced our statistical power.

Fourth, analyses that compared survey respondents and nonrespondents indicated some small but significant differences in baseline characteristics: As noted above, women and people with higher earnings were more likely to respond to the survey.^27^

A fifth limitation was that our outcome of interest was self-reported.

Sixth, the data were available for thirty-two months after randomization, a relatively short time frame. It can take time for improved economic outcomes to translate into measurable health benefits.^32^ The short follow-up period was compounded by the young age and overall good health of the participants.

## Study Results

The Paycheck Plus plan provides adults without dependent children with a credit of up to $2,000 annually and expands eligibility up to annual earnings of $30,000. Under the federal EITC, adults without dependent children are eligible for a maximum credit of just over $500 (about 15 percent of the maximum for one-child families), and the credit phases out at very low income levels (about $15,000) (exhibit 1).

The socioeconomic outcomes of Paycheck Plus have been reported elsewhere.^27,31^ Among participants eligible for the bonus in the treatment group, 65 percent received it in the first year. However, this declined to 58 percent in the second year and 57 percent in the third year. The share of the treatment group that got the bonus was 46 percent in the first year, 35 percent in the second year, and 30 percent in the third year. On average, participants in the treatment group who received a bonus received an additional $1,400 per year. Participation in the program increased after-bonus earnings by 6 percent over the three years, which corresponds to an increase of $635 per year. This amount is modest for most adults, but it can be significant for those with very few financial resources. Paycheck Plus reduced the incidence of severe poverty by 3.4 percentage points but had no effect on material hardship or the overall poverty rate. Over the three-year period, the program increased the annual employment rate by 1.9 percentage points, on average. Effects on employment rates were larger among women and more disadvantaged men. The program had no effects on secondary social outcomes such as marital status and living arrangements or criminal justice involvement.

At baseline, 59 percent of the participants were male, and over half were age thirty-five or younger (exhibit 2). Over 80 percent were either Hispanic or non-Hispanic black. Less than a quarter of the sample had attended college, and 18 percent had been incarcerated in the past. Forty-five percent were working, but only 24 percent were working thirty hours or more per week. Twenty-nine percent did not have any earnings in the past year. Sixty-one percent had filed a tax return in the previous tax year, but less than half of the sample had heard of the EITC. Only 19 percent had received the EITC in the past year. There were no significant differences between the treatment and control groups at baseline, which indicates that randomization was successful.

Overall, respondents were in good health, with mean EQ5D-5L scores of 0.94 and 0.95 in the control and treatment groups, respectively (exhibit 3). Eligibility for the program did not have an effect on health-related quality of life at thirty-two months after randomization. Respondents also reported low levels of limitations across the five domains that compose the overall score, ranging from 1.13 to 1.59. Consistent with the overall score, we did not find significant differences between the groups in terms of limitations in mobility, self-care, usual activities, pain/discomfort, or anxiety/depression.

Heterogenous effects by sex are reported in exhibit 4 for the overall score and by domains. Stratified analyses did not show significant differences between men and women. However, women who were eligible for Paycheck Plus had higher gains in EQ5D-5L scores than men. As women had lower health-related quality of life (appendix exhibit 5),^26^ these results indicate that eligibility for the program reduced inequalities in health-related quality of life by sex. The predicted mean health-related quality of life for eligible women was 0.99, a 0.05-point difference from the average score of women in the control group. When we turned to domains of the score, we found that a reduction in limitations with usual activities drove the overall improvement in health-related quality of life (*p*< 0.001 for the interaction term).

## Discussion

Two important findings emerged from the first thirty-two months of the Paycheck Plus experiment. First, a sizable expansion of EITC benefits for adults without dependent children was associated with modest increases in income, earnings, and work. The modest economic impacts, combined with the young age and good health of the participants, suggest that any secondary effects on health-related quality of life are likely to be small. Second, positive effects on health did emerge for women. The reduction in inequalities in health-related quality of life by sex was consistent with the larger effects of Paycheck Plus on employment and earnings among women than among men.

A number of factors might explain these modest effects on health-related quality of life. First, Paycheck Plus was associated with just a 6 percent increase in income, on average, across the full sample (including those who did not receive the bonus), but it did reduce the incidence of severe poverty. Its effects on employment took time to appear and remained modest in the second and third years of the program. These effect sizes are in line with prior research on how employment rates respond to tax credits for people who are employed.^11^

Second, not all respondents who were eligible for the credit ended up filing tax returns and thereby claiming the credit. While uptake was lower than that of the federal EITC for working families, it was similar to that of adults without dependent children.31 Therefore, the effects we present correspond to eligibility for Paycheck Plus but not to receipt of the expanded EITC per se. However, even if we rescaled the estimates by uptake, they would remain modest.

A third and related factor is that of the duration of follow-up available at the New York City site. Income and employment interventions may produce changes in depression over a short period, because mental health states can change rapidly,

However, it is more difficult to affect self-rated health or other physical health outcomes in the short term. The modest effects found in this study might be a result of this short time frame and are consistent with the existing quasi-experimental literature that documented small or null effects of the EITC in the short term versus larger impacts in the longer run.^24^

Finally, an important dimension of the existing EITC related to socioeconomic and health outcomes is that it is fully integrated into the tax system and conditional on employment.^33^ As these features of the program have been shown to lower the stigma usually attached to receiving welfare benefits and to affect the way the credit is spent,^34^ future research should examine how the enrollment and dispersal mechanisms of Paycheck Plus potentially affect outcomes differently. For example, a demonstration in Chicago, Illinois, has shown that periodic payments instead of a lump sum were associated with a reduction in food insecurity among low-income working families.^35^

These findings add to the limited experimental literature on the effect of antipoverty programs on health in the United States.^32^ One example of experimental social policy research is the negative income tax experiments conducted in the 1970s across the US. Like Paycheck Plus, these experiments tested the effect of increases in tax credits for lower-income Americans. However, while the negative income tax provided larger payments to unemployed Americans than to those with earnings, Paycheck Plus encourages low-income families to work and, for those with low earnings, to increase their earnings. This is important because income and employment are thought to work in tandem to improve health, with employment also providing social capital.^36^ For treated participants in the negative income tax experiments, the benefits of increased income might be canceled out by the harms of lower employment. Those experiments were associated with modest or no health impacts.^37,38^

In the 1990s the US government experimentally tested the effect of imposing time limits (typically five years) on welfare receipt that did not depend on income. These experiments showed positive impacts on income and employment and a large decline in the number of welfare recipients. However, they were also linked to increases in mortality, potentially among families whose members were unable to find work and ended up having their cash benefits cut off at the end of the five-year eligibility period.^39^ A model of conditional cash transfers has also been tested in New York (the Family Rewards program), which provided cash assistance on the condition of engaging in activities that promoted human capital and health such as school attendance, employment, and accessing health care. Family Rewards improved socioeconomic outcomes and reduced poverty but was associated with modest health benefits among adults and no effects among children.^40^

We found that the beneficial effects of the intervention we studied were larger among women than men, for both employment and quality-of-life outcomes. These findings are in line with quasi-experimental evidence that the short-term effects of receiving the EITC on a range of health indicators were more beneficial to the health of women than that of men.^24^ This effect has been seen in other evaluations of the effect of social policy on health as well.^41^ It is worth noting that the positive effect of Paycheck Plus on the health of women was primarily realized via their ability to perform usual activities. These include work, housework, and family or leisure activities that are linked to health. Recall that the EQ5D-5L is a combined measure of five health domains, with mobility, self-care, pain/discomfort, and anxiety/depression accompanying usual activities. Although the domains of the EQ5D-5L scale have not been validated as separate measures, this result provides valuable information on the mechanism through which the expanded credit might affect household consumption and health-related quality of life. Qualitative evidence on the EITC indicates that recipients tend to view and spend the lump sum differently than they do their usual income.^42,43^ They also use the lump sum to make larger purchases and invest in durable goods.^44^ How this translates into health improvements in the longer term should be the subject of future research.

Our analysis of Paycheck Plus provides much-needed evidence on the policy options available to reduce the strong association between poverty and poor health, using a credible causal design. These results can also inform the development and evaluation of similar tax credits for workers across other high-income countries. As noted in a systematic review of the health effects of these programs, the existing body of evidence had a high risk of bias, and limited conclusions could be drawn on that basis.^45^ The Paycheck Plus evaluation fills this gap and highlights the importance of rigorous experimental evaluation of social programs and their effect on health.

## Policy Implications

The federal and state governments have recently pushed for a greater emphasis on the social determinants of health.^46^ The hope is that addressing upstream determinants will improve health and potentially reduce spending. The EITC has emerged as a tool to decouple income and health and ultimately reduce socioeconomic inequalities in health. We present the first experimental evidence on an extension of the program to workers without dependent children, a population group left out of expansions of the 1990s. The intervention was associated with modest reductions in extreme poverty and, subsequently, modest improvements in health-related quality of life among women. As the expansion has bipartisan support, it has the potential to be enacted. While pending confirmation with longer follow-up and clinical data, our study provides some optimism that reshaping the social policy landscape could reverse the declining health of low-income Americans observed in recent years.

## Supplementary Material

Supplemental Appendix

## Figures and Tables

**Exhibit 1:**
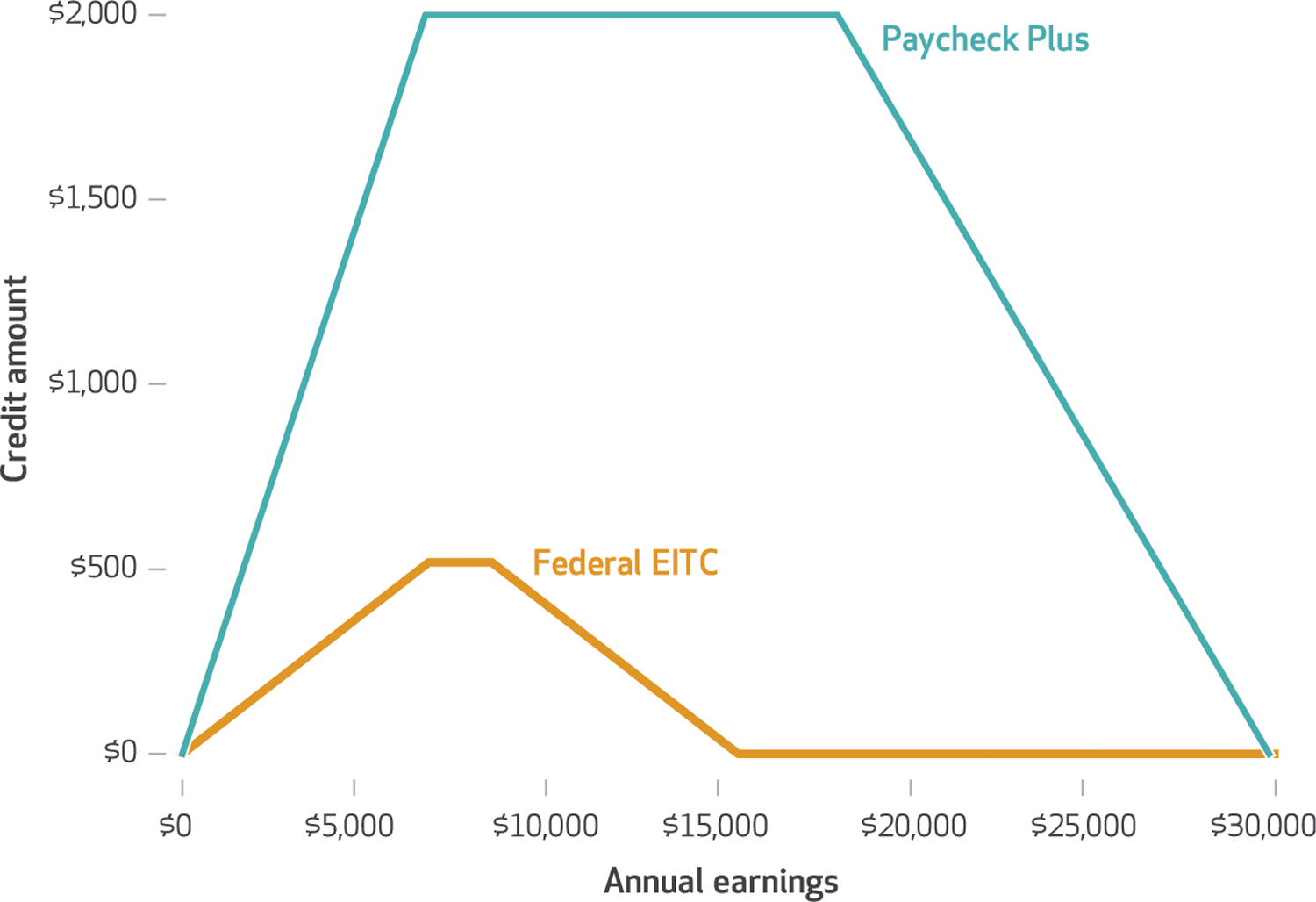
Earned Income Tax Credit (EITC) amounts based on annual earnings for the Paycheck Plus treatment group and control group in New York City, 2018 **SOURCE** Authors’ analysis of data on EITC parameters from the Tax Policy Center (2017); and data from Miller C, et al. Final impact findings from the Paycheck Plus demonstration in New York City (see note 27 in text). **NOTES** Credit amounts are received by participants when they file income tax returns, based upon their annual earnings. The control group received the federal EITC. The line labeled “Federal EITC” illustrates the credit amount for a single adult in the control group with no qualifying children, based on annual earnings. Phase-in and phase-out rates are calculated as set percentages of earnings. As a low-income household earns more, its credit increases (“phase-in”) until it reaches a first threshold. The credit stays constant at the maximum amount until it reaches a second threshold, when the amount decreases (“phase-out”) until it reaches zero. The phase-in and phase-out rates for the federal EITC shown are 7.65 percent. The phase-in rate for Paycheck Plus is 30.0 percent, and the phase-out rate is 17.0 percent.

**Exhibit 2:** Selected baseline characteristics of participants in the Paycheck Plus program in New York City, overall and by treatment or control group, 2014

Characteristics	Overall (*N* = 5,968)	Treatment (*n* = 2,997)	Control (*n* = 2,971)
Male	59.0%	58.3%	59.8%
Age, years			
35 or younger	53.0	54.1	52.0
Older than 35	47.0	45.9	48.0
Race/ethnicity			
Hispanic	30.0	29.6	30.4
Non-Hispanic black	57.8	57.9	57.6
Non-Hispanic white	12.2	12.5	11.9
Education			
High school diploma or equivalent	54.0	52.7	55.3
Some college	24.2	25.3	23.2
Ever incarcerated	18.1	17.2	18.9
Currently working	45.2	45.4	44.9
Working full time^a^	23.8	23.5	24.1
Earnings in the past year			
$0	29.4	29.9	29.0
$1–$6,666	28.2	27.9	28.4
$6,667–$17,999	29.4	29.4	29.4
$18,000 or more	13.0	12.7	13.2
Filed a tax return in previous tax year	60.7	60.6	60.8
Has heard of the EITC	45.8	45.9	45.7
Has received the EITC in the past	19.0	18.7	19.3

**SOURCE** Authors’ analysis of Paycheck Plus baseline survey data. **NOTES** The baseline sample included respondents recruited between September 2013 and February 2014. To assess differences in characteristics across intervention groups, we used *t*-tests and chi-square tests. EITC is Earned Income Tax Credit.

a Working thirty hours or more per week.

**Exhibit 3:** Effect of Paycheck Plus on health-related quality of life in New York City, by control or treatment group, thirty-two months after randomization

	Control (*n* = 1,701)	Treatment (*n* = 1,588)	Adjusted difference
Overall EQ5D-5L score	0.94	0.95	0.01
Domain score			
Mobility	1.38	1.36	−0.01
Self-care	1.14	1.13	0.01
Usual activities	1.33	1.32	0.01
Pain/discomfort	1.59	1.59	0.01
Anxiety/depression	1.45	1.39	−0.04

**SOURCE** Authors’ analysis of Paycheck Plus baseline and thirty-two-month survey data. A randomly selected subset of the baseline sample (*n* = 3,289) received the thirty-two-month survey. **NOTES** The control and treatment columns present unadjusted means. Adjusted differences are the differences between the groups, obtained from Poisson regressions. All models controlled for age, sex, education level, race/ethnicity, earnings in the year before enrollment in Paycheck Plus, history of incarceration, and timing of data collection. The full results of the effect of Paycheck Plus on health-related quality of life are in appendix exhibit5 (see note 26 in text). The EQ5D-5L score (explained in the text) ranges from 0 to 1.

**Exhibit 4:** Effect of Paycheck Plus on health-related quality of life in New York City, by sex and control or treatment group, thirty-two months after randomization

	Women			Men			
	Control (*n* = 701)	Treatment (*n* = 781)	Adjusted difference	Control (*n* = 870)	Treatment (*n* = 897)	Adjusted difference	Interaction
Overall EQ5D-5L score	0.94	0.95	0.01	0.95	0.94	−0.01	0.02**
Domain score							
Mobility	1.41	1.36	−0.02	1.36	1.36	0.01	−0.03
Self-care	1.15	1.13	−0.01	1.13	1.13	0.00	−0.01
Usual activities	1.37	1.29	−0.05	1.30	1.34	0.04	−0.09***
Pain/discomfort	1.64	1.60	−0.01	1.55	1.59	0.03	−0.05
Anxiety/depression	1.49	1.42	−0.05	1.42	1.37	−0.03	−0.02

**SOURCE** Authors’ analysis of Paycheck Plus baseline and thirty-two-month survey data. **NOTES** A randomly selected subset of the baseline sample (*n* = 3,289) received the thirty-two-month survey. The control and treatment columns present adjusted differences (see the notes to exhibit 3). Variables controlled for in the models and the EQ5D-5L score are explained in the notes to exhibit 3. The interaction column presents coefficients for the interaction between treatment and female sex. Full results of the effect of Paycheck Plus by sex are in appendix exhibit 5 (see note 26 in text).

** *p* < 0:05

*** *p* < 0:01
