# Supplementary material for: The Health Effects Of Expanding The Earned Income Tax Credit: Results From New York City
Source: Health Aff (Millwood). Author manuscript; Available in PMC 2021 Feb 26. (PMC7909715; doi:10.1377/hlthaff.2019.01556)
Supplement: Supplemental Appendix [file NIHMS1668740-supplement-Supplemental_Appendix.pdf]

Courtin E, Aloisi K, Miller C, Allen HL, Katz LF, Muennig P. The health effects of expanding the earned income tax credit: results from New York City. Health Aff (Millwood). 2020;39(7).

# APPENDIX

**Appendix Exhibit 1.** Timing of the Paycheck Plus demonstration in New York City

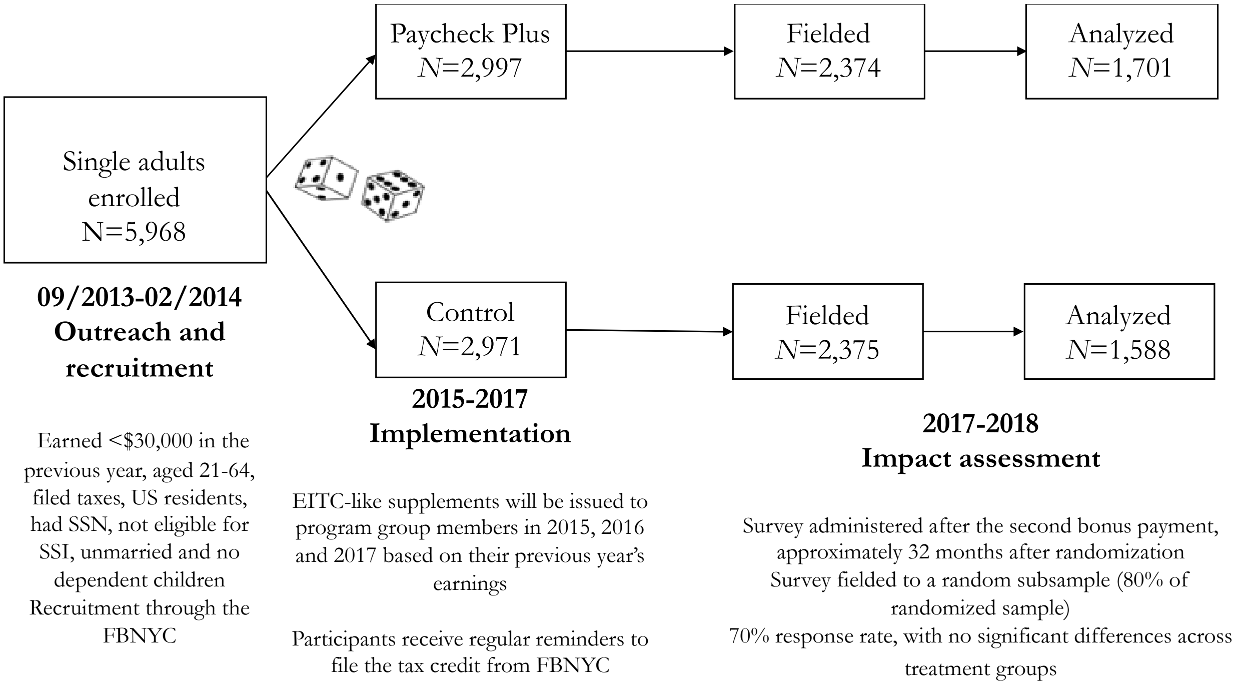


**Source**: Adapted from Miller et al. 2018.

**Notes:** EITC: Earned Income Tax Credit; SSN: Social Security Number; SSI: Supplemental Security Information; FBNYC: Food Bank for New York City.

**Appendix Exhibit 2.** Inclusion flow diagram, Paycheck Plus at New York City site

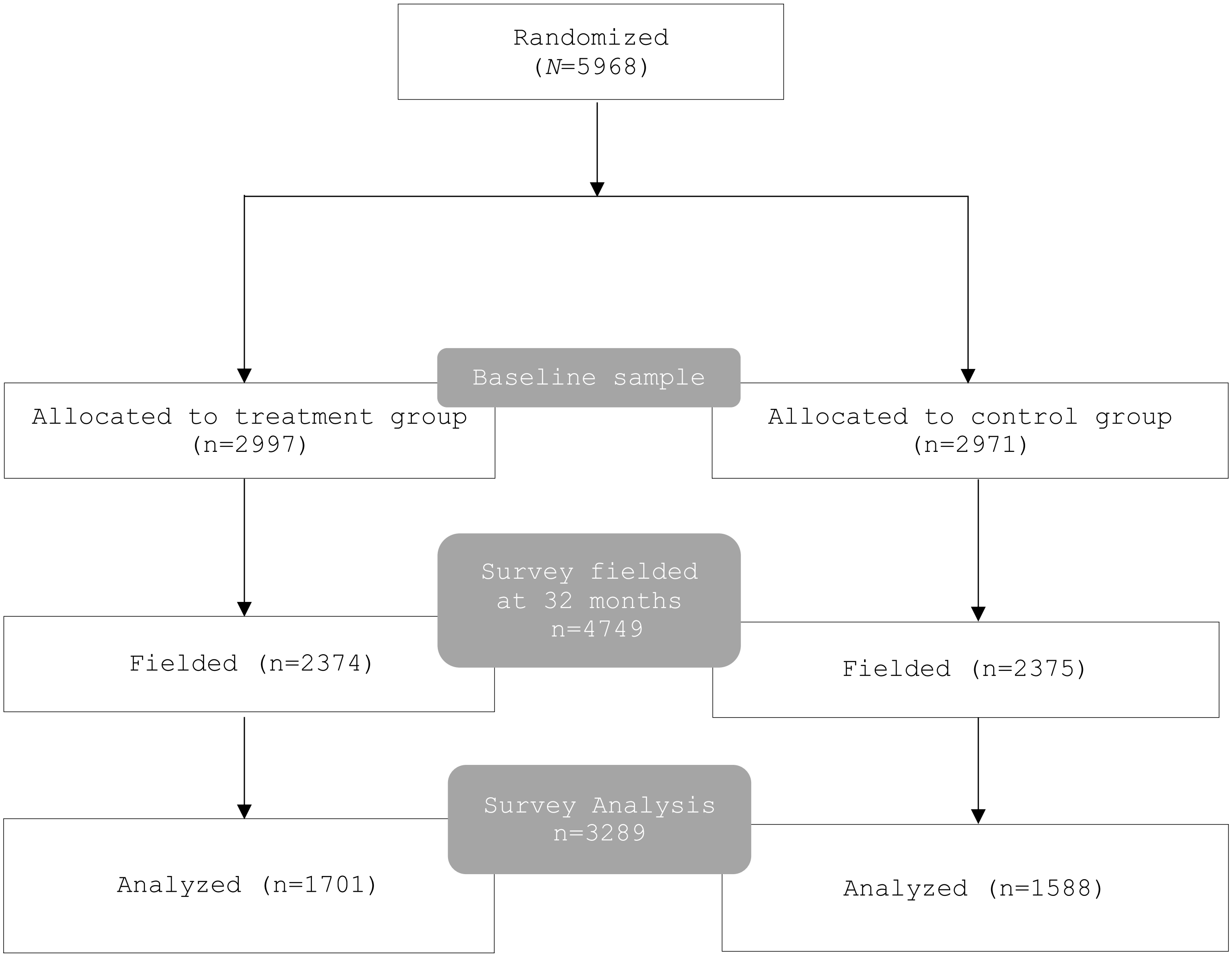

**Appendix Exhibit 3.** Effect of Paycheck Plus on health-related quality of life estimated using ordinary least square models, overall sample and by gender, Paycheck Plus at New York City site

| | **Overall sample** | | **Women** | | **Men** | | **Interaction** | |
|---|---|---|---|---|---|---|---|---|
| | Estimate | *P*-value | Estimate | *P*-value | Estimate | *P*-value | Estimate | *P*-value |
| Eligibility to Paycheck Plus | 0.005 | 0.289 | 0.01 | 0.059 | 0.00 | 0.282 | -0.007 | 0.330 |
| Eligibility*female | - | - | - | - | - | - | 0.021 | 0.054 |
| Employed at baseline | 0.01 | <0.001 | 0.025 | 0.003 | 0.028 | 0.002 | 0.02 | 0.001 |
| Black | 0.007 | 0.272 | 0.010 | 0.379 | 0.033 | 0.012 | 0.031 | <0.001 |
| Hispanic | -0.016 | 0.039 | -0.014 | 0.259 | 0.0003 | 0.982 | -0.009 | 0.343 |
| Aged 25 to 34 (ref. 21 to 24) | 0.001 | 0.829 | 0.003 | 0.764 | -0.009 | 0.430 | -0.008 | 0.314 |
| Aged 35 to 44 | -0.031 | <0.001 | -0.034 | 0.004 | -0.048 | <0.001 | -0.051 | <0.001 |
| Aged 45 or older | -0.047 | <0.001 | -0.046 | <0.001 | -0.079 | <0.001 | -0.078 | <0.001 |
| Female | -0.009 | 0.059 | - | - | - | - | -0.029 | <0.001 |
| Ever incarcerated | -0.002 | 0.793 | -0.006 | 0.481 | -0.007 | 0.484 | -0.005 | 0.528 |
| Earnings in the prior 3 quarters | -0.000 | 0.312 | -0.000 | 0.244 | -0.000 | 0.646 | -0.000 | 0.9213 |
| Number of quarters employed in the prior 3 quarters | 0.009 | 0.003 | 0.012 | 0.004 | 0.005 | 0.005 | 0.010 | 0.001 |
| Noncustodial parent | 0.006 | 0.475 | 0.011 | 0.248 | 0.015 | 0.149 | 0.010 | 0.125 |
| Has GED/high school diploma or higher (ref. less than GED/high school diploma) | -0.004 | 0.578 | -0.006 | 0.424 | -0.010 | 0.268 | -0.002 | 0.785 |

**Sources**: Paycheck Plus baseline and 32-month survey data. The 32-month survey is on a randomly selected subset of the baseline sample (*N*=3,289).

**Notes:** GED: General Educational Development. All models also adjust for month of random assignment. The overall sample columns include all respondents. The next two columns present the effect of the program, stratified by gender. The last columns display the estimates from a model including an interaction term between the treatment (eligibility to Paycheck Plus) and gender (female).

**Appendix Exhibit 4.** Distribution of the EQ5D-5L outcome

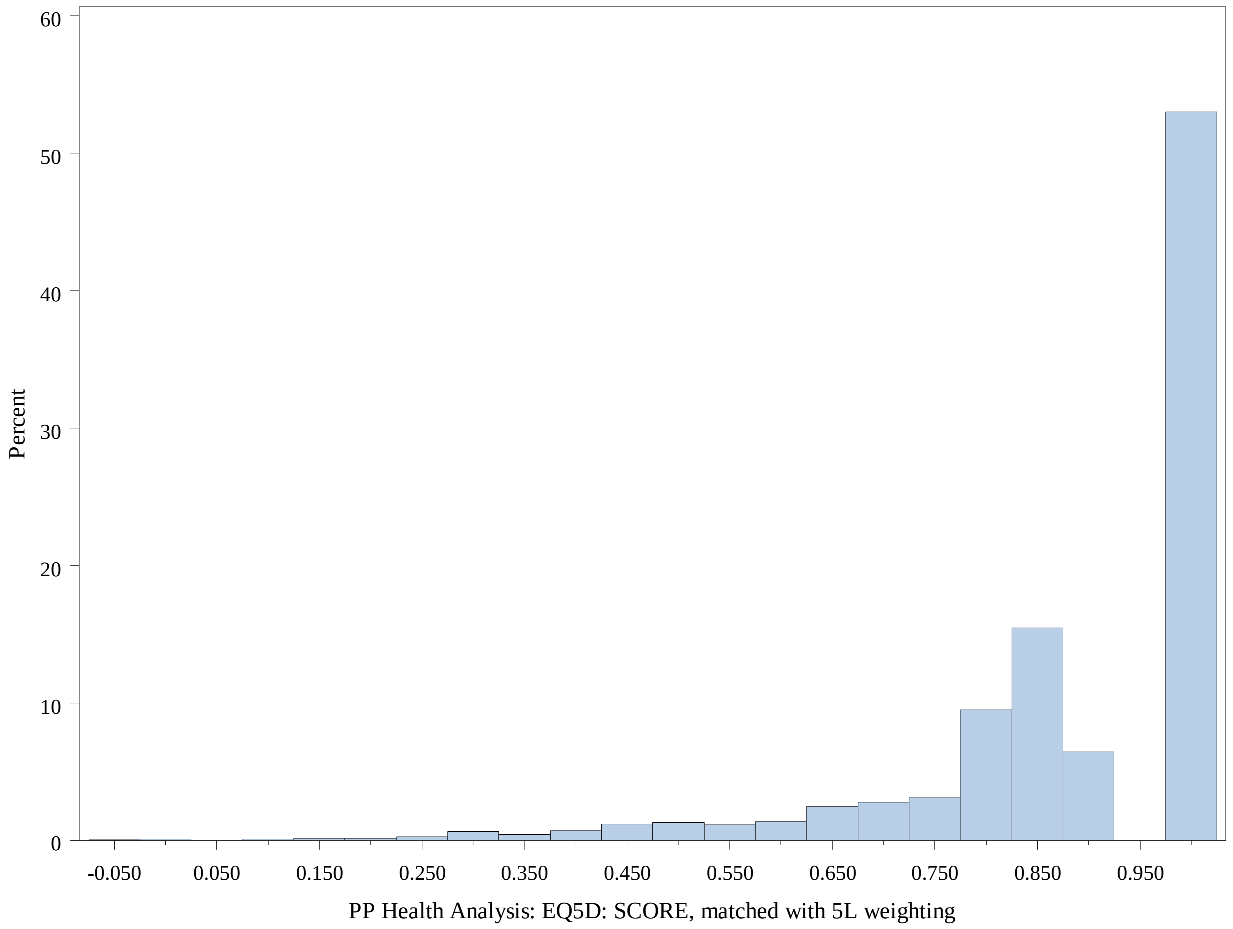


**Sources**: Paycheck Plus 32-month survey data. The 32-month survey is on a randomly selected subset of the baseline sample (*N*=3,289). Negative values correspond to 'worse than death' values.

**Appendix Exhibit 5**. Effect of Paycheck Plus on health-related quality of life estimated using Poisson regressions, overall sample and by gender

| | **Overall sample** | | **Women** | | **Men** | | **Interaction** | |
|---|---|---|---|---|---|---|---|---|
| | Estimate | *P*-value | Estimate | *P*-value | Estimate | *P*-value | Estimate | *P*-value |
| Eligibility to Paycheck Plus | 0.01 | 0.289 | 0.01 | 0.070 | -0.01 | 0.635 | -0.008 | 0.319 |
| Eligibility*female | - | - | - | - | - | - | 0.02 | 0.049 |
| Employed at baseline | 0.02 | <0.001 | 0.016 | 0.045 | 0.022 | 0.001 | 0.023 | <0.001 |
| Black | 0.007 | 0.272 | 0.012 | 0.201 | 0.004 | 0.694 | -0.012 | 0.253 |
| Hispanic | -0.016 | 0.039 | -0.022 | 0.064 | -0.011 | 0.285 | -0.012 | 0.253 |
| Aged 25 to 34 (ref. 21 to 24) | 0.001 | 0.829 | -0.001 | 0.984 | 0.003 | 0.701 | -0.009 | 0.236 |
| Aged 35 to 44 | -0.031 | <0.001 | -0.032 | 0.019 | -0.031 | 0.004 | -0.057 | <0.001 |
| Aged 45 or older | -0.047 | <0.001 | -0.04 | <0.001 | -0.048 | <0.001 | -0.087 | <0.001 |
| Female | -0.009 | 0.059 | - | - | - | - | -0.035 | <0.001 |
| Ever incarcerated | -0.002 | 0.792 | 0.004 | 0.836 | -0.002 | 0.755 | -0.007 | 0.498 |
| Earnings in the prior 3 quarters | -0.000 | 0.312 | -0.000 | 0.748 | -0.000 | 0.311 | -0.000 | 0.950 |
| Number of quarters employed in the prior 3 quarters | 0.009 | 0.003 | 0.006 | 0.172 | 0.012 | 0.010 | 0.012 | 0.004 |
| Noncustodial parent | 0.006 | 0.475 | 0.024 | 0.153 | -0.012 | 0.110 | 0.017 | 0.139 |
| Has GED/high school diploma or higher (ref. less than GED/high school diploma) | -0.004 | 0.092 | 0.011 | 0.363 | -0.012 | 0.091 | -0.003 | 0.716 |

**Sources**: Paycheck Plus baseline and 32-month survey data. The 32-month survey is on a randomly selected subset of the baseline sample (*N*=3,289).

**Notes:** GED: General Educational Development. All models also adjust for month of random assignment. The overall sample columns include all respondents. The next two columns present the effect of the program, stratified by gender. The last columns display the estimates from a model including an interaction term between the treatment (eligibility to Paycheck Plus) and gender (female).
